# A Soft Sensor Modeling Method Based on Local Migration Modeling Framework

**DOI:** 10.3390/s25237182

**Published:** 2025-11-25

**Authors:** Bo Wang, Shaowen Huang, Hangfei Cai

**Affiliations:** Key Laboratory of Agricultural Measurement and Control Technology and Equipment for Mechanical Industrial Facilities, School of Electrical and Information Engineering, Jiangsu University, Zhenjiang 212013, China; 2222307043@stmail.ujs.edu.cn (S.H.); 2222207107@stmail.ujs.edu.cn (H.C.)

**Keywords:** transfer learning, soft sensor modeling, deep neural networks, firefly optimization algorithms

## Abstract

To address the issues of low model fitting accuracy and insufficient prediction capability caused by the multi-stage characteristics and batch-to-batch data distribution heterogeneity in the fermentation process of *Pichia pastoris*, this study proposed a novel soft sensor modeling method with deep transfer learning (DTL) strategies to propose a novel soft sensor modeling method based on a local transfer modeling framework. Fermentation process data were partitioned into multiple sub-source domains using the K-means clustering algorithm. For each sub-source domain, Deep Neural Networks (DNNs) were employed to establish prediction models, which were further optimized using an improved firefly algorithm. The Euclidean distance between the target domain samples and the cluster centroids of each sub-source domain was calculated to perform correlation analysis and identification. The sub-source domain with the highest correlation to the target domain samples was selected, and a deep transfer fine-tuning method was applied to optimize the corresponding sub-source domain model, ultimately obtaining the target domain prediction model. The experimental results indicated that the proposed method extracts local feature information from fermentation process data, enhancing prediction accuracy and model generalization performance. This provides a viable approach for soft sensor modeling in multi-condition fermentation scenarios of *Pichia pastoris* fermentation processes.

## 1. Introduction

*Pichia pastoris* has become a key platform for the production of exogenous proteins, and is widely used in various fields such as biomedicine, cosmetic skincare, and industrial enzyme preparations [1]. However, the highly nonlinear and time-varying characteristics with strong coupling of the *Pichia pastoris* fermentation process makes it difficult to directly measure some key biochemical parameters online. Accurate measurement of these parameters is essential for optimizing fermentation processes and improving product quality. Therefore, as an indirect measurement method, soft sensor technology has been extensively researched and applied in predicting the fermentation process of *Pichia pastoris* [2].

Traditional soft sensor methods primarily include linear models such as Partial Least Squares (PLS) and nonlinear models like Artificial Neural Networks (ANNs) [3,4,5]. Wang (2025) highlighted the effectiveness of PLS in addressing multi-variable correlation in batch processes but noted its inadequacy in capturing nonlinear relationships [6]. With the advancement of machine learning, ANNs and deep learning variants (e.g., Deep Neural Networks, DNNs) have been widely adopted in fermentation soft sensors due to their strong nonlinear fitting capabilities [7,8]. However, most of these methods have relied on global modeling frameworks, having assumed uniform data distribution throughout the fermentation cycle—an assumption that has contradicted the actual characteristics of *Pichia pastoris* fermentation. Specifically, *Pichia pastoris* fermentation is characterized by multi-phase features, including the lag, exponential, stationary, and decline phases, and data distributions vary across these phases [9]. Global models trained on full-cycle data often result in the averaging of average local phase information, resulting in reduced fitting accuracy for individual phases.

To address the multi-phase issue, several local modeling strategies have been proposed. Zhou, Y. et al. (2023) developed a phase-based soft sensor for fed-batch fermentation using fuzzy C-means (FCM) clustering for phase division and Support Vector Regression (SVR) for sub-modeling, and achieved higher accuracy than global models [10]. However, these local methods only focused on phase partitioning within a single batch and failed to address batch-to-batch data distribution heterogeneity—a critical challenge in *Pichia pastoris* fermentation. Variations in medium composition, inoculum concentration, and operating conditions often result in significant distribution differences between batches. This heterogeneity causes trained models to exhibit poor generalization when applied to new target batches [11].

Transfer Learning (TL) has emerged as a promising approach to address cross-domain distribution shifts by leveraging knowledge from data-rich source domains to improve learning in target domains [12,13,14,15]. In recent years, Deep Transfer Learning (DTL), which integrates deep learning’s feature extraction ability with TL’s knowledge transfer capability, has been introduced into fermentation soft sensors. For example, Li et al. (2024) proposed a DTL-based soft sensor for DO concentration in industrial fermentation, and achieved better performance than traditional models by transferring pre-trained deep models between batches [16,17]. However, most existing DTL-based methods adopt global transfer frameworks, transferring knowledge from the entire source domain to the target domain without considering the multi-phase local characteristics of fermentation. This inconsistency between global transfer and local phase features limited the extraction of phase-specific information and restricts further performance improvement [17].

Additionally, optimization of local sub-models and selection of relevant source domains require further refinement [18]. Heuristic optimization algorithms, such as the Firefly Algorithm (FA), have demonstrated considerable efficacy in soft sensor parameter optimization owing to their robust global search capabilities [19,20], but the application of improved FA in optimizing local sub-models remains underexplored. For source domain selection, Euclidean distance is widely used for batch data similarity analysis [21], yet its integration into a local transfer framework to select the most relevant sub-source domain has not been systematically studied.

To address these research gaps, this study aims to develop a high-performance soft sensor method for *Pichia pastoris* fermentation that simultaneously handles multi-phase characteristics and batch-to-batch heterogeneity. A local transfer modeling framework is proposed by integrating local modeling theory and DTL (deep transfer learning strategies): (1) K-means clustering divided fermentation data into multiple phases, and partitions the training set into sub-source domains; (2) DNN-based sub-source domain models were established, with an improved FA which was used to optimize DNN hyperparameters to enhance local fitting accuracy; (3) Euclidean distance was computed to measure the similarity between target samples and sub-source domain centroids, to select the most relevant sub-source domain; and (4) DTL fine-tuning was applied to adapt the optimal sub-source domain model to the target domain, yielding the final prediction model.

Experimental results demonstrated that the proposed method effectively extracted local phase features and alleviated batch-to-batch heterogeneity. Compared with traditional global models and existing local and transfer learning methods, it exhibits significantly higher prediction accuracy and generalization performance. This work provided a new solution for *Pichia pastoris* fermentation soft sensor under multi-operational conditions and serves as a framework for addressing similar challenges in other microbial fermentation processes.

Theoretically, it integrates local modeling and deep transfer learning for the first time to address the dual challenges of multi-phase characteristics and batch heterogeneity in *Pichia pastoris* fermentation, thereby addressing limitations in existing soft sensor approaches. Practically, the proposed method enables the effective extraction of local phase features and transfer domain adaptive knowledge, leading to improved prediction accuracy and generalization performance. It provides a new technical solution for real-time monitoring of key biochemical parameters in industrial *Pichia pastoris* fermentation processes under multi-operating conditions, which can support the dynamic optimization of fermentation processes and reduce production costs, thereby ensure product quality stability—thus promoting the intelligent advancement of microbial fermentation industries.

## 2. Theoretical Analysis

### 2.1. Multi-Model Local Modeling Framework Based on K-Means Clustering

Conventional global modeling approaches treat the entire fermentation dataset as a homogeneous unit, which fails to capture the distinct metabolic patterns of *Pichia pastoris* fermentation—specifically, the cell proliferation phase, fed-batch transition phase, and protein induction phase. To address this limitation, a *K*-means clustering-based phase division strategy is employed to partition the historical source batch data into phase-specific sub-source domains, laying the foundation for subsequent local model training. The core of *K*-means clustering lies in minimizing the Within-Cluster Sum of Squares (WCSS), which quantifies the cohesion of data points within each cluster. The WCSS is mathematically defined as:

(1) $$J=\sum_{i=1}^{K}\sum_{x\in C_{i}}{\left\|x-\mu_{i}\right\|}^{2}$$

where K denote the number of clusters; $C_{i}$ represent the set of data points comprising the i-th cluster; x signify a data point $C_{i}$; $\mu_{i}$ correspond to the centroid of the i-th cluster, calculated as $\mu_{i}=\frac{1}{C_{i}}\sum_{x\in C_{i}}x$; and ${\left\|x-\mu_{i}\right\|}^{2}$ is the squared Euclidean distance between data point x and cluster centroid $\mu_{i}$,with the Euclidean distance itself defined as:

(2) $$d_{i}={\left\|x-\mu_{i}\right\|}^{2}=\sqrt{\sum_{j=1}^{n}{\left(x_{j}-\mu_{i,j}\right)}^{2}}$$

where n represents the number of features (or dimensions) in the dataset, $x_{j}$ is the *j*-th feature of x, and $\mu_{i,j}$ is the *j*-th component of $\mu_{i}$.

During the clustering process, as the number of clusters increases, the partition of data samples becomes more refined, which leads to higher intra-cluster cohesion. Consequently, the sum of squared errors (SSE, a pivotal metric in the elbow method) gradually decreases. The SSE is defined as:

(3) $$SSE={\sum_{i=1}^{K}\sum_{p\in C_{i}}\left|p-m_{i}\right|}^{2}$$

Let $C_{i}$ denote the i-th cluster, p represent the set of all data points belonging to, $C_{i}$ and $m_{i}$ be the centroid of $C_{i}$. When the number of clusters k is set below the true number of underlying groups, increasing k significantly enhances the intra-cluster cohesion of each partition. Consequently, the decline rate of the error metric SSE; when the number of clusters k reaches the true number of underlying groups, further increments in k yield rapidly diminishing returns in intra-cluster cohesion. Consequently, the marginal decrease in the error metric SSE sharply attenuates and gradually plateaus as k continues to increase beyond the true cluster count. The relationship plot between the number of clusters k and the error SSE exhibits an “elbow” shape, where the inflection point on the curve corresponds to the true number of clusters in the data.

**Figure 1:**
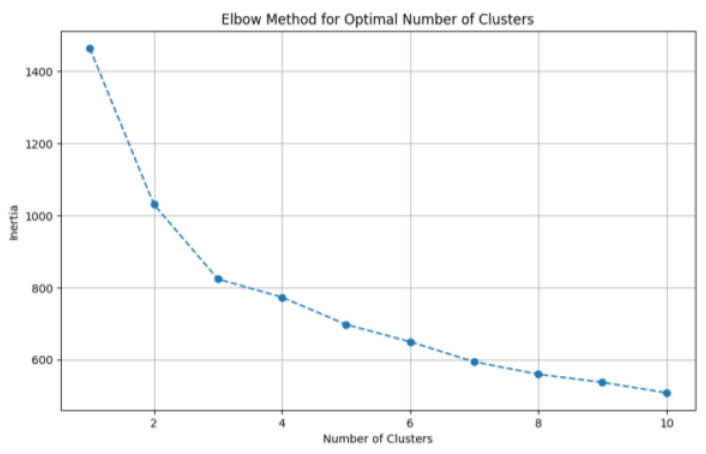
Elbow method plot.

**Figure 2:**
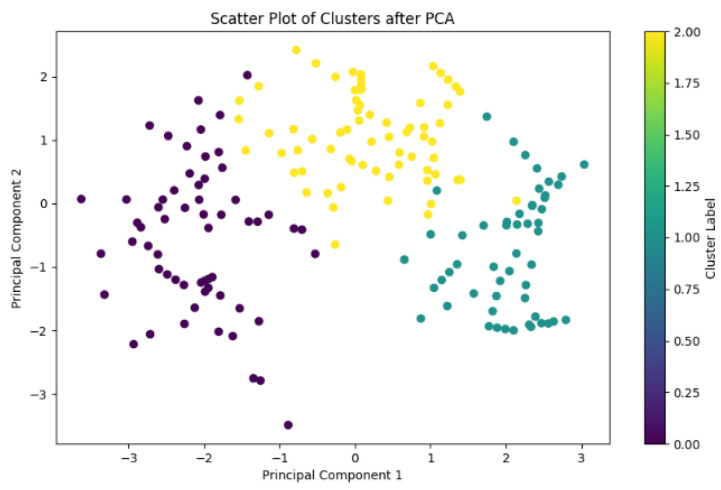
Cluster distribution plot.

The elbow method was applied to a dataset of *Pichia pastoris* samples after dimensionality reduction via Principal Component Analysis (PCA). The resulting elbow plot, where the vertical axis (inertia) represents the within-cluster sum of squared errors (SSE) was illustrated in Figure 1. As depicted in the plot, the maximum curvature occurs at k = 3 clusters, corresponding to the “elbow” identified by the elbow method. Consequently, this paper selected k = 3 as the optimal number of clusters for *K*-means clustering. Figure 2 presents the scatter plot of PCA reduced data partitioned into three clusters, each corresponding to one fermentation phase. These three clusters were further designated as sub-source domains *D*_1_, *D*_2_, and *D*_3_ for subsequent local model training.

For a given target batch sample, its Euclidean distance to the centroid of each sub-source domain was computed using Equation (2). The sub-source domain with the smallest distance was identified as the most relevant with the target sample, and the corresponding local model was selected as the pretrained model for transfer learning.

### 2.2. Sub-Source Domain Prediction Modeling Using Deep Neural Networks

In soft sensor applications for *Pichia pastoris* fermentation, deep neural network (DNN) modeling exhibits powerful learning and nonlinear approximation capabilities to precisely uncover intricate relationships within fermentation data. This enables real-time, high-precision prediction of key biochemical parameters, thereby providing robust data support for the optimization and control of fermentation processes.

Deep Neural Network (DNN), a cornerstone technology machine learning, construct artificial neural network models by emulating the structural and functional architectures of biological neuronal networks in the human brain. These models consist of multiple interconnected layers of artificial neurons.

When categorized by their positions within a Deep Neural Network (DNN), the internal layers of a DNN can be classified into three types: the input layer, the hidden layer (s), and the output layer.

Given an input X, the weight connecting node i in layer l to node j in layer l − 1 is denoted as $w_{ij}^{\left(l\right)}$. The bias term for node i in layer l is $b_{i}^{\left(l\right)}$. The network’s output is $y_{m}$, and both the hidden layers and the output layer employ the sigmoid activation function.

The computational process of deep neural networks consists of two fundamental components: forward propagation and backward propagation. The forward propagation phase can be formally represented as follows:

The output of the j-th neuron in the l-th layer is mathematically expressed as follows:

(4) $$y_{j}^{l}=f\left(\sum_{k}w_{jk}^{l}a_{k}^{l-1}+b_{j}^{l}\right),$$

The forward propagation equations for a layer in a Deep Neural Network (DNN) can be compactly expressed in matrix form. Given:

(5) $$y^{l}=f\left(w^{l}a^{l-1}+b^{l}\right)$$

The forward propagation process iteratively computes layer-wise outputs using Equation (5), culminating in the model’s final prediction, However, forward propagation alone only yields the model’s predicted outputs; it does not update the model parameters. Model parameters are updated by backpropagating the error between predictions (from forward pass) and ground truth labels (from experimental data).

Let Y = {$y_{1}$, $y_{2}$, …, $y_{m}$} denote the ground truth. The loss function is defined as the mean squared error (MSE) between the model’s predictions and ground truth labels:

(6) $$E\left(w,b\right)=\frac{1}{2}\sum_{k=1}^{n}{\left(y_{k}-y_{k}^{l}\right)}^{2}=\frac{1}{2}\sum_{k=1}^{n}{\left(y_{k}-f_{w,b}(x_{k})\right)}^{2}$$

Parameters are updated using a gradient descent-based optimization method, according to the general update rule:

(7) $$x\leftarrow x-\eta \nabla f\left(x\right)$$

where $f\left(x\right)$ is a function of parameter x, ∇ denotes the gradient operator, and η represents the learning rat.

Based on the above derivations, the gradient descent algorithm for updating parameters in deep neural networks is defined as

(8) $$\left\{\begin{array}{l} w_{ij}^{\left(l\right)}\leftarrow w_{ij}^{\left(l\right)}-\eta \nabla \omega_{ij}^{\left(l\right)}\\ b_{i}^{\left(l\right)}\leftarrow b_{i}^{\left(l\right)}-\eta \nabla b_{i}^{\left(l\right)} \end{array}\right.$$

where $\nabla \omega_{ij}^{\left(l\right)}=\frac{\partial E\left(w,b\right)}{\partial \omega_{ij}^{l}}$, $\nabla b_{i}^{\left(l\right)}{=b}_{i}^{\left(l\right)}-\eta \frac{\partial E\left(w,b\right)}{\partial b_{i}^{l}}$.

The calculated weights are and bias update:

(9) $$\left\{\begin{array}{l} w_{ij}^{\left(l\right)}=w_{ij}^{\left(l\right)}+\eta \nabla \omega_{ij}^{\left(l\right)}\\ b_{i}=b_{i}+\eta \nabla b_{i}^{\left(l\right)} \end{array}\right.$$

At this stage, the backpropagation phase is completed, and a deep neural network model with a predicted output closer to the ground truth is trained.

### 2.3. Model Optimization via Improved Firefly Algorithm

The performance of DNN models is highly dependent on hyperparameters (e.g., initial weights, learning rate, number of hidden neurons). The traditional Firefly Algorithm (FA)—a nature-inspired metaheuristic—tends to suffer from premature convergence to local optima when optimizing DNN hyperparameters [19]. To address this, an Improved Firefly Algorithm (IFA) was developed by incorporating a random perturbation strategy, Differential Evolution (DE)-based crossover, and selection mechanism.

#### 2.3.1. Traditional FA Fundamentals

In FA, each firefly is encoded as a candidate solution vector (representing DNN hyperparameters) [22]. The brightness of a firefly is proportional to its fitness value, defined as the reciprocal of the DNN’s MSE (Equation (6)):

(10) $$I_{i}=\frac{1}{1+L(W_{i},B_{i})}$$

where *I_i_* is the brightness of the *i*-th firefly, and $W_{i},B_{i}$ are the DNN parameters corresponding to the *i*-th firefly.

The attractiveness between firefly *i* and firefly *j* (where $I_{j}\;>\;I_{i}$) is:

(11) $$\beta_{i,j}(r)=\beta_{0}e^{-\gamma r_{i,j}^{2}}$$

where $\beta_{0}$ is the maximum attractiveness at *r* = 0, γ is the light absorption coefficient, and $r_{i,j}$ is the Cartesian distance between firefly *i* and *j* in the solution space:

(12) $$r_{i,j}=\sqrt{\sum_{d=1}^{D_{s}}{\left(x_{i,d}-x_{j,d}\right)}^{2}}$$

where *D_S_* is the dimension of the solution space (number of DNN hyperparameters), and *x_i_*_,*d*_, *x_j_*_,*d*_ are the d-the components of the *i*-th and *j*-the firefly vectors, respectively.

Firefly *i* moves toward firefly *j* (brighter firefly) as follows:

(13) $$x_{i,d}^{\left(t+1\right)}=x_{i,d}^{\left(t\right)}+\beta_{i,j}(r)(x_{j,d}^{\left(t\right)}-x_{i,d}^{\left(t\right)})+\alpha \epsilon_{d}$$

where α is the step size (0<α<1), and $\epsilon_{d}$ is a random number following a uniform distribution *U*(0,1).

#### 2.3.2. Improved Firefly Algorithm

(1) stochastic perturbation strategy.

To avoid local optima, a random perturbation was added to the updated position of each firefly [23]:

(14) $$x_{i,d}^{(t+1),\mathrm{pert}}=x_{i,d}^{\left(t+1\right)}+\delta (x_{p,d}^{\left(t\right)}-x_{q,d}^{\left(t\right)})$$

where δ~U(0,1) is the perturbation factor, and p, q are randomly selected firefly indices (distance from *i*).

(2) DE-Based Crossover.

Fireflies were crossed with the current global best firefly ($x_{\mathrm{best},d}$) to refine optimal solutions. The crossover probability was proportional to the firefly’s fitness:

(15) $$P_{i}=\frac{I_{i}}{\sum_{k=1}^{N_{f}}I_{k}}$$

where $N_{f}$ is the number of fireflies. The crossover operation generated a new solution:

(16) $$x_{i,d}^{(t+1),\mathrm{cross}}=\left\{\begin{array}{ll} x_{\mathrm{best},d}^{\left(t\right)} & \mathrm{if}\;U(0,1)<P_{i}\\ x_{i,d}^{(t+1),\mathrm{pert}} & \mathrm{otherwise} \end{array}\right.$$

(3) Levy Flight.

To enhance global search capability, Levy Flight was integrated into the position update [24,25,26]. The step size of Levy Flight follows a Levy distribution with probability density function:

(17) $$L(s;\lambda )=\frac{\lambda \Gamma (\lambda )\sin(\pi \lambda /2)}{\pi }s^{-(\lambda +1)}\;\;\;\;(0<\lambda <2)$$

where Γ(⋅) is the gamma function. The step size *s* was generated using the Mantegna algorithm, and the position update was adjusted to:

(18) $$x_{i,d}^{\left(t+1\right)}=x_{i,d}^{(t+1),\mathrm{cross}}+s\cdot \mathrm{sign}(U(0,1)-0.5)$$

**Figure 3:**
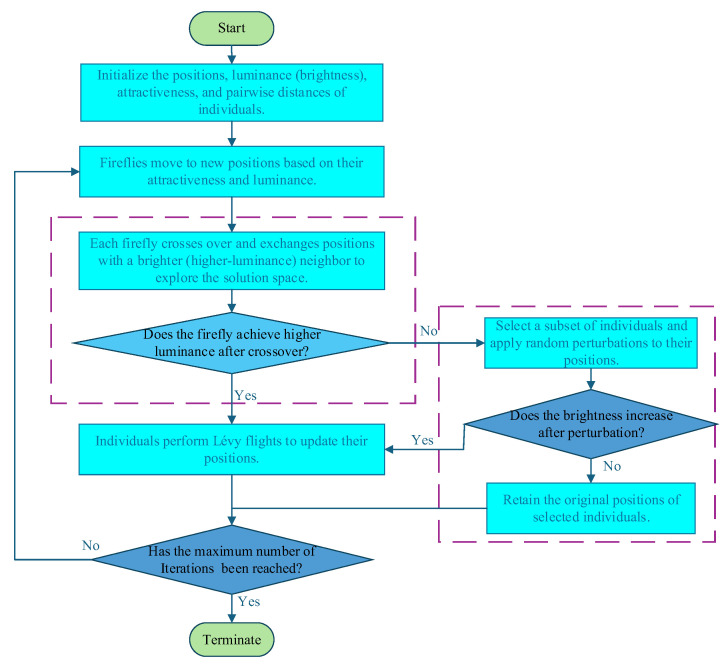
Flowchart of the Improved Firefly Algorithm.

The IFA optimization process terminates when the maximum number of iterations is reached or the fitness value converges. The optimal firefly vector is decoded to derive the DNN hyperparameters for each sub-source domain model. A flowchart of the IFA is provided in Figure 3 and Algorithm 1.
**Algorithm 1:** Parameter Settings of the Improved Firefly Algorithm.**Input:** Population size n, Max iterations T, Objective function f(x) **Output:** Global best solution ${g\;}^{*}$
1.  **Initialize** firefly population $\left\{x_{1},x_{2},...,x_{n}\right\}$ randomly2.  Compute brightness $I_{i}=1/f(x_{i})$ for each firefly i3.  ${g\;}^{*}\leftarrow arg\;max\;I_{i}$ // Initialize global best4.  **for** t=1 **to** T **do**5.        **for** i=1 **to** n **do**6.              **for** j=1 **to** n **do**7.                    **if** $I_{j}>I_{i}$ **then**8.                          Compute distance $r_{ij}=∥x_{i}-x_{j}∥$9.                          Compute attractiveness $\beta =\beta_{0}\cdot e^{-\gamma r_{ij}^{2}}$10.                        $x_{i}\leftarrow x_{i}+\beta \cdot (x_{j}-x_{i})+\alpha \cdot s$11.                        $x_{i}\leftarrow x_{i}+\eta \cdot (x_{r1}-x_{r2})$12.                        Update brightness $I_{i}$13.                        **if** $I_{i}>I(g^{*})$ **then** $g^{*}\leftarrow x_{i}$14.                  **end if**15.            **end for**16.      **end for**17.      **for** i=1 **to** n **do**18.            **if** $rand()<P_{crossover}(I_{i})$ **then**19.                  $x_{i}^{new}\leftarrow x_{i}+P_{crossover}\cdot (g^{*}-x_{i})$20.                  **if** $f(x_{i}^{new})<f(x_{i})$ **then** $x_{i}\leftarrow x_{i}^{new}$ // Greedy selection21.            **end if**22.      **end for**23.**end for**24.**return** $g^{*}$

### 2.4. Deep Transfer Learning for Batch Heterogeneity Mitigation

During the fermentation process of *Pichia pastoris*, variations in operational conditions across different fermentation batches lead to differences in data distribution during the fermentation. However, since these batches essentially represent the same reaction process, models trained on data from different batches must share certain similarities. Therefore, useful information learned from other fermentation batches can be utilized to assist the target fermentation batch in completing its tasks [14].

**Figure 4:**
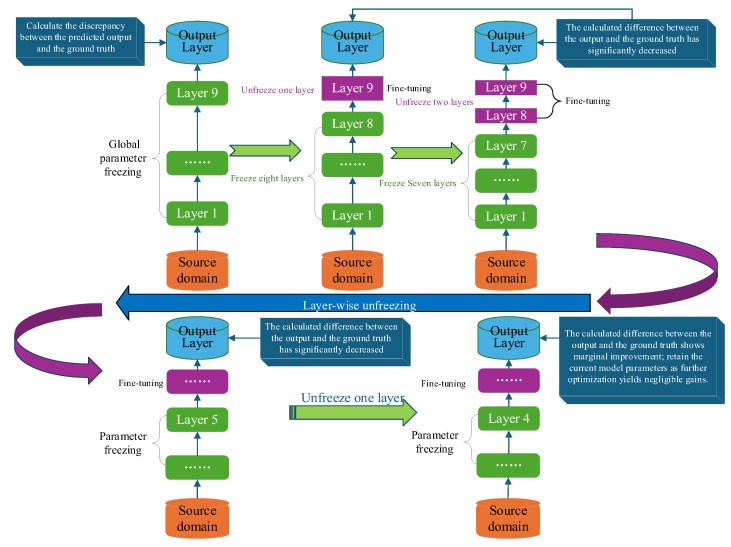
Structure-Based Transfer Learning.

Transfer learning applies the model architecture knowledge learned from an old task (source task) to a new task (target task). Even when the target task has a limited dataset, structure-based transfer learning can achieve more accurate predictions by transferring structural knowledge from the source task. In transfer learning, it is essential to first address the issue of data probability distribution in the datasets, followed by employing strategies such as freezing and fine-tuning [26] (see Figure 4).

Due to the highly nonlinear characteristics of *Pichia pastoris* fermentation data, which implies more complex data distribution discrepancies between source and target domains, it is necessary to minimize the Maximum Mean Discrepancy (MMD) metric to reduce the divergence in data probability distributions.

Assuming the source domain data $D_{S}={\left\{X_{i}^{S}\right\}}_{i=1}^{n_{S}}$ and the target domain data $D_{T}={\left\{X_{j}^{T}\right\}}_{j=1}^{n_{T}}$, this paper use a deep neural network $f_{\theta }\left(•\right)$ as the feature extractor, we obtain source domain features $z_{i}^{S}=f_{\theta }\left(X_{i}^{S}\right)$ and target domain features $z_{j}^{T}=f_{\theta }\left(X_{j}^{T}\right)$, the Maximum Mean Discrepancy (MMD) is defined as:

(19) $$L_{MMD\left(D_{S},D_{T}\right)}={\left\|\frac{1}{n_{S}}\sum_{i=1}^{n_{S}}z_{i}^{S}-\frac{1}{n_{T}}\sum_{j=1}^{n_{T}}z_{j}^{T}\right\|}^{2}$$

Through the optimization of $\underset{\theta }{\min}\;L_{MMD\left(D_{S},D_{T}\right)}$, the parameters θ in DNN $f_{\theta_{new}}\left(•\right)$ are updated to enhance the model’s predictive accuracy for target domain data.

After obtaining $f_{\theta_{new}}\left(•\right)$, freezing and fine-tuning operations are performed as illustrated in Figure 1. The freezing strategy preserves most or all pre-trained model parameters during transfer learning. The depth of the neural network was optimized prior to the main experiments. We conducted a grid search over architectures with 5 to 11 hidden layers and found that a 9-hidden-layer network yielded the lowest validation loss. This empirically determined architecture is employed herein, with the first k layers initially frozen to facilitate knowledge transfer from the source domains. Training begins with all layers locked, followed by sequential layer-by-layer unfreezing. The process stops when unfreezing additional layers fails to boost validation set performance beyond a preset threshold.

(20) $$\theta_{i}^{\left(t+1\right)}=\left\{\begin{array}{c} {\theta_{i}}^{\left(t\right)}-\eta_{forzen}\cdot \nabla_{\theta_{i}}L\;\;\;\;\;i>k\\ {\theta_{i}}^{\left(t\right)}-\eta_{finetune}\cdot \nabla_{\theta_{i}}L\;\;\;\;\;i>k \end{array}\right.$$

where ${\theta_{i}}^{\left(t\right)}$ denotes the parameters of the i-th layer at the t-th iteration, $\theta_{i}^{\left(t+1\right)}$ represents the parameters of the i-th layer at the (t+1)-th iteration, $\eta_{forzen}$ stands for the extremely small learning rate applied to frozen layers, $\eta_{finetune}$ indicates the maximum learning rate for fine-tuning layers, and $\nabla_{\theta_{i}}L$ signifies the gradient of the loss function with respect to the parameters. The parameter set $\theta_{i}=\left\{w_{i},b_{i}\right\}$ comprises both weights and biases, and the loss function is defined as follows:

(21) $$L=\frac{1}{N}\sum_{n=1}^{N}{\left(y_{pred}^{\left(n\right)}-y_{true}^{\left(n\right)}\right)}^{2}$$

where $y_{pred}$ is the predicted value of the model, and $y_{true}$ is the true value.

Fine-tuning Strategy refers to the selective updating of a subset of parameters (typically the topmost or final layers) in a pre-trained model while keeping other layers frozen during task-specific training.

The fine-tuning process for frozen layers proceeds as follows:

(22) $$\left\{\begin{array}{l} w_{forzen}\leftarrow w_{forzen}-\alpha_{forzen}\cdot \frac{\partial L}{\partial w_{forzen}}\\ b_{forzen}\leftarrow b_{forzen}-\alpha_{forzen}\cdot \frac{\partial L}{\partial b_{forzen}} \end{array}\right.$$

where $w_{forzen}$ represents the weights of the fine-tuning layer, $\alpha_{forzen}$ denotes an extremely small learning rate applied to the frozen layers, while $\frac{\partial L}{\partial w_{forzen}}$ and $\frac{\partial L}{\partial b_{forzen}}$ are the gradients of the loss function L with respect to the weights and biases, respectively.

(23) $$\left\{\begin{array}{l} w_{finetune}\leftarrow w_{finetune}-\alpha_{finetune}\cdot \frac{\partial L}{\partial w_{finetune}}\\ b_{finetune}\leftarrow b_{finetune}-\alpha_{finetune}\cdot \frac{\partial L}{\partial b_{finetune}} \end{array}\right.$$

where $w_{finetune}$ denotes the weights of the fine-tuning layer; $\alpha_{finetune}$ represents an exceptionally large learning rate applied to the frozen layers; while $\frac{\partial L}{\partial w_{finetune}}$ and $\frac{\partial L}{\partial b_{finetune}}$ correspond to the gradients of the loss function L with respect to the weights and biases, respectively.

### 2.5. Transfer Learning Modeling Based on K-IFA-DNN

**Figure 5:**
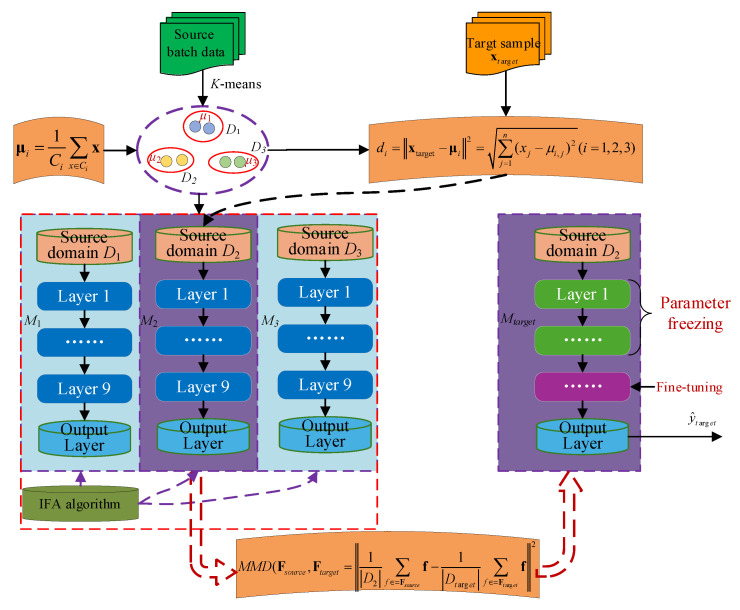
**Algorithm Design Flowchart.** Note: The framework integrates *K*-means clustering for phase division, IFA-DNN for sub-model training, and deep transfer learning for target domain adaptation, forming a closed loop for handling multi-phase and batch-heterogeneous fermentation data.

The IFA-DNN sub-models trained on phase-specific sub-source domains exhibit strong fitting capabilities for historical source batches. However, batch-to-batch heterogeneity in *Pichia pastoris* fermentation (e.g., variations in raw material purity or seed culture activity) results in distribution shifts between source and target batch data. This mismatch reduces the generalization capability of pre-trained sub-models when directly applied to target batches. To address this, a local transfer learning framework was proposed, which leverages the phase-specific knowledge of IFA-DNN sub-models and adapts it to the target domain via layer-wise freezing and fine-tuning. This integration ensures the model retains phase-specific feature extraction capabilities while adapting to batch-wise distribution differences. The complete workflow is illustrated in Figure 5, with detailed steps as follows:

Step 1: **Sub-Source Domain Partitioning:** Historical source batch data was clustered into 3 sub-source domains (*D*_1_, *D*_2_, *D*_3_) using K-means (Section 2.1). For each sub-domain, the cluster centroid was calculated using $\mu_{i}=\frac{1}{C_{i}}\sum_{x\in C_{i}}x$ and stored as *μ*_1_, *μ*_2_, *μ*_3_. These centroids serve as phase-feature benchmarks for subsequent target sample matching.

Step 2: **IFA-DNN Sub-Model Training:** For each D*_k_* (*k* = 1, 2, 3) an IFA-DNN sub-model *M_k_* was constructed via the DNN architecture and IFA hyperparameter optimization (Section 2.2 and Section 2.3), yielding three pre-trained sub-models *M*_1_, *M*_2_, *M*_3_.

Step 3: **Target Sub-Source Domain Selection:** For a given target sample $x_{t\arg et}$ (with auxiliary variables measured but key biochemical parameters unknown), using the Euclidean distance formula compute the distance between $x_{t\arg et}$ and each stored centroid ($d_{i}={\left\|x_{\mathrm{target}}-\mu_{i}\right\|}^{2}=\sqrt{\sum_{j=1}^{n}{\left(x_{j}-\mu_{i,j}\right)}^{2}}(i=1,2,3)$). Identify the sub-source domain with the smallest distance $d_{min}=\min(d_{1},d_{2},d_{3})$ (e.g., *M*_2_). The corresponding IFA-DNN sub-model (*M*_2_ in this case) was selected as the base transfer model, as its trained phase features (e.g., fed-batch transition phase) are most aligned with $x_{\mathrm{target}}$-ensuring transferred knowledge is relevant to the target sample’s metabolic state.

Step 4: **Deep Transfer Fine-Tuning:** The base model *M*_2_ was fine-tuned using limited labeled target domain data $D_{\mathrm{target}}$, minimizing distribution mismatch between *D*_2_ (source) and $D_{\mathrm{target}}$ (target) ($MMD(F_{source},F_{target}={\left\|\frac{1}{\left|D_{2}\right|}\sum_{f\in =F_{source}}f-\frac{1}{\left|D_{\mathrm{target}}\right|}\sum_{f\in =F_{\mathrm{target}}}f\right\|}^{2}$), and applying layer-wise freezing/fine-tuning (Section 2.4) to obtain the target-adapted model $M_{target}$.

Note: For feature extracted by the DNN’S hidden layers (denoted as $F_{source}$ from *D*_2_ and $F_{\mathrm{target}}$ from $D_{\mathrm{target}}$.

Step 5: **Target Sample Prediction:** The optimized model $M_{target}$ was applied to $x_{\mathrm{target}}$ to predict the key biochemical parameter value ${\hat{y}}_{\mathrm{target}}$ via forward propagation (Equation (4)).To validate the framework’s effectiveness, the prediction performance was evaluated using metrics including Root Mean Squared Error (RMSE) and Coefficient of Determination (R^2^), benchmarked against traditional method (e.g., global DNN, non-optimized FA-DDN) to confirm improvements in accuracy and generalization.

(24) $$RMSE=\sqrt{\frac{1}{n}\sum_{i=1}^{n}{\left(y_{pre}^{\left(i\right)}-{\hat{y}}_{real}^{\left(i\right)}\right)}^{2}}$$

(25) $$R^{2}=1-\frac{\sum_{i=1}^{n}{\left(y_{pre}^{\left(i\right)}-y_{real}^{\left(i\right)}\right)}^{2}}{\sum_{i=1}^{n}{\left(y_{real}^{\left(i\right)}-{\hat{y}}_{real}\right)}^{2}}$$

## 3. Emulation

To validate the proposed soft sensor modeling method based on the local transfer framework for addressing the multi-stage characteristics and batch-to-batch data heterogeneity in *Pichia pastoris* fermentation, a systematic emulation has been designed, including data preprocessing (to provide high-quality input for modeling) and model construction (to implement the local transfer logic). The detailed methodology is outlined below:

### Data Preprocessing

(1) Data acquisition.

Sample data are collected at 4-h intervals during the fermentation process. Environmental parameters (e.g., temperature, pH, dissolved oxygen) and input variables (e.g., feed flow rates) are automatically measured using built-in instrumentation or sensors integrated with the bioreactor. These measurements are recorded ten times within the four-hour interval and subsequently converted into a 4-h average using the difference quotient method. In contrast, key biochemical parameters—including product concentration (inulinase activity), *Pichia pastoris* biomass concentration, and methanol concentration—are determined through off-line laboratory assays conducted every 4 h (i.e., coinciding with the 4-h sampling timepoints).

(2) Increase the amount of data.

Given the scarcity of experimental data with only three batches available, each containing 61 data points, it is necessary to augment the dataset using a method that increases the number of internal samples for training the soft sensor model, thereby improving prediction accuracy. This study employs linear interpolation as the data augmentation technique. Linear interpolation involves constructing an interpolant as a first-degree polynomial, ensuring zero interpolation error at the given data nodes. The augmentation strategy inserts two new data points between each pair of adjacent original data points, effectively expanding each batch from 61 to 181 total points and generating 120 additional interpolated samples per batch. These interpolated samples are partitioned into two distinct subsets of 60 points each for model. Compared to higher-order methods (e.g., quadratic interpolation), linear interpolation offers simplicity and computational efficiency, making it a pragmatic choice for dataset expansion in resource-constrained scenarios while maintaining data consistency between auxiliary and target variables.

(26) $$y=y_{0}+\frac{y_{1}-y_{0}}{x_{1}-x_{0}}\times \left(x-x_{0}\right)$$

where x and y denote the horizontal and vertical coordinates of the interpolated data points, while $x_{0}$, $y_{0}$, $x_{1}$, $y_{1}$ represent the coordinates of two adjacent original data points used for interpolation.

(3) Dimensionality reduction (selection of auxiliary variables).

Given the high dimensionality of the measured dataset due to the multitude of features, the direct use of raw data for model construction often results in inaccurate or unstable models owing to the curse of dimensionality. To address this challenge, this study employs Principal Component Analysis (PCA) as a dimensionality reduction technique. PCA is a linear transformation method that projects high-dimensional data onto a lower-dimensional subspace while preserving the dominant variance structures and eliminating redundant or correlated features. By doing so, PCA facilitates the extraction of latent principal components that encapsulate the most informative patterns in the data, thereby enhancing model interpretability and generalization performance.

(27) $$X=(X_{1},X_{2},\cdots ,X_{p})$$

where $X_{j}$ (where 1≤j≤p) denotes a column vector representing the values of the j-th feature across all data points in the dataset.

The mean vector of the calculated sample is:

(28) $$\bar{x}=\frac{1}{p}\sum_{i=1}^{p}x_{i}$$

The dataset is centered by subtracting the mean value from each feature (column vector), such that for every feature, X the centered feature $\bar{x}$ is computed as:

(29) $$X_{centered}=X-\bar{x}$$

The covariance matrix of the dataset is computed as follows:

(30) $$\Sigma =\frac{1}{p}X_{centered}^{T}X_{centered}$$

The covariance matrix is subjected to eigenvalue decomposition, yielding eigenvalues $\lambda_{1},\;\lambda_{2}$ ⋯⋯ $\lambda_{n}$ and their corresponding eigenvectors $v_{1},v_{2}$ ⋯⋯ $v_{n}$.

The top *k* eigenvectors corresponding to the largest eigenvalues are selected as principal components, forming a projection matrix W where each column represents an eigenvector. The dataset is projected onto the subspace spanned by the top *k* eigenvectors, yielding a low-dimensional representation of the data. Mathematically, the projection is computed as:

(31) $$X_{reduced}=X_{centered}W$$

where $X_{reduced}$ denotes the dimension-reduced dataset.

**Figure 6:**
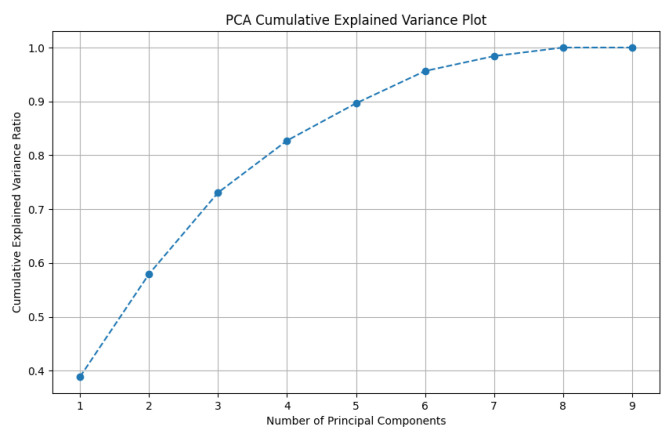
Principal Component Analysis.

For determining the number of principal components to retain, the cumulative explained variance method can be employed. This approach quantifies the total variance proportion explained by the first *n* principal components, reflecting their collective ability to represent the data. Its purpose is to guide the selection of *n* such that most of the information in the original dataset is preserved. The calculation involves summing the individual explained variance ratios of each principal component. An example of this is illustrated in Figure 6, which depicts the cumulative explained variance plot for PCA.

As depicted in Figure 6, the selection of 8 principal components from the original set of 9 features achieved an optimal balance, yielding the highest cumulative explained variance. This dimensionality reduction from 9 to 8 components successfully retained the most critical information from the original dataset. Consequently, this optimization streamlined the model by reducing computational overhead and resource consumption while simultaneously enhancing its predictive accuracy and generalization performance.

## 4. Simulation Results

To systematically validate the effectiveness of the proposed local transfer modeling framework in addressing the multi-stage characteristics and batch-to-batch data heterogeneity of *Pichia pastoris* fermentation, simulation experiments were designed to evaluate two core components: (1) the performance of local sub-source domain models (established via K-means clustering and improved firefly algorithm-optimized DNN) and (2) the predictive capability of the target domain model (obtained via deep transfer fine-tuning). The models were trained on a system equipped with an Intel Core i5 CPU and a Max 250 GPU. The hyperparameter optimization using our Improved Firefly Algorithm required approximately 10 min per run. Key biochemical parameters—Pichia yeast concentration (ug/mL), Inulinase concentration (U/mL), and Methanol concentration (g/L)—were selected as evaluation metrics, with results analyzed in detail below.

### 4.1. Performance Evaluation of Local Sub-Source Domain Models

The primary goal of this section was to verify whether partitioning the source domain into homogeneous sub-domains (via K-means) and optimizing DNN models with an improved firefly algorithm (IFA) enhanced the fitting accuracy of local fermentation stage characteristics (e.g., lag phase, exponential growth phase, stationary phase).

**Figure 7:**
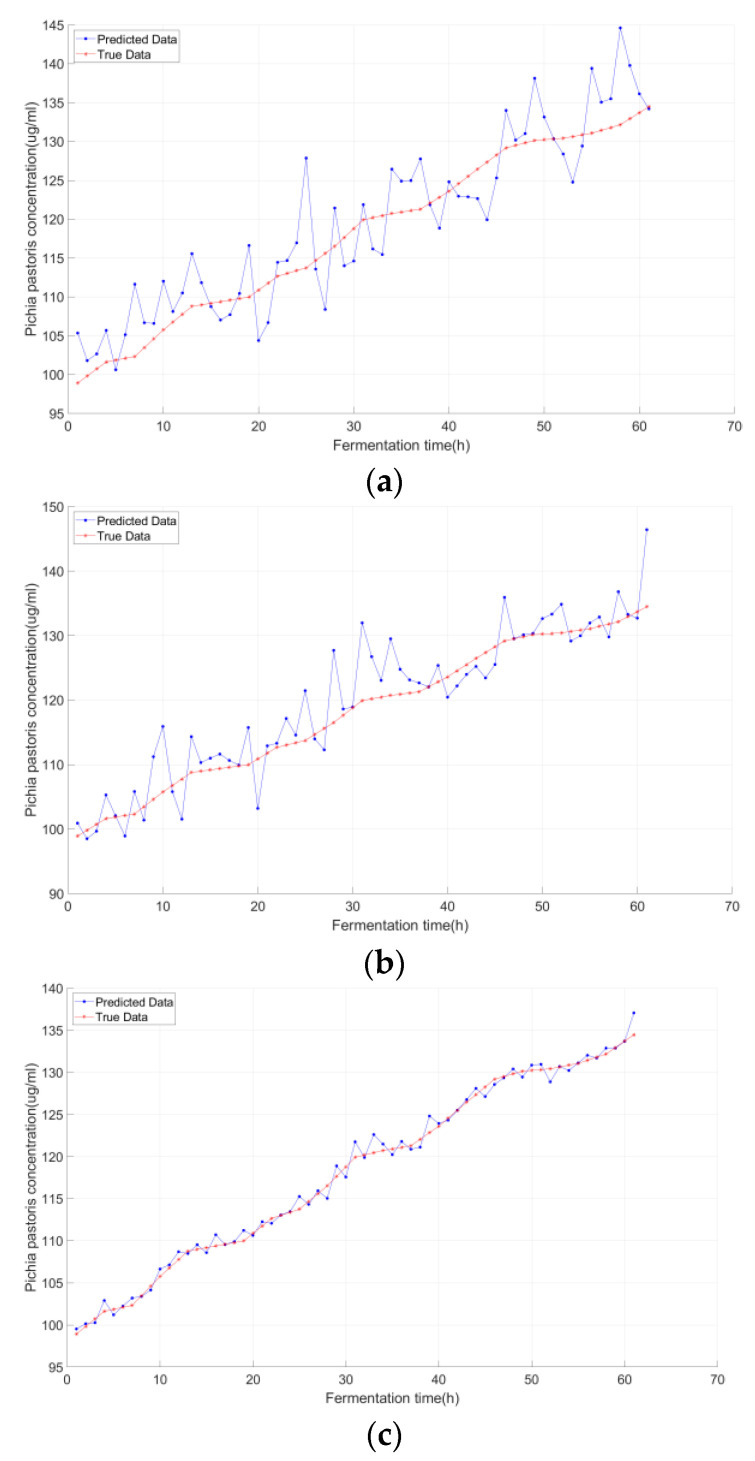
***Pichia pastoris* concentration predictions under different modeling approaches:** (**a**) Deep Neural Network (DNN); (**b**) DNN optimized by the conventional Firefly Algorithm (FA-DNN); (**c**) Proposed Model (K-FA-DNN).

**Figure 8:**
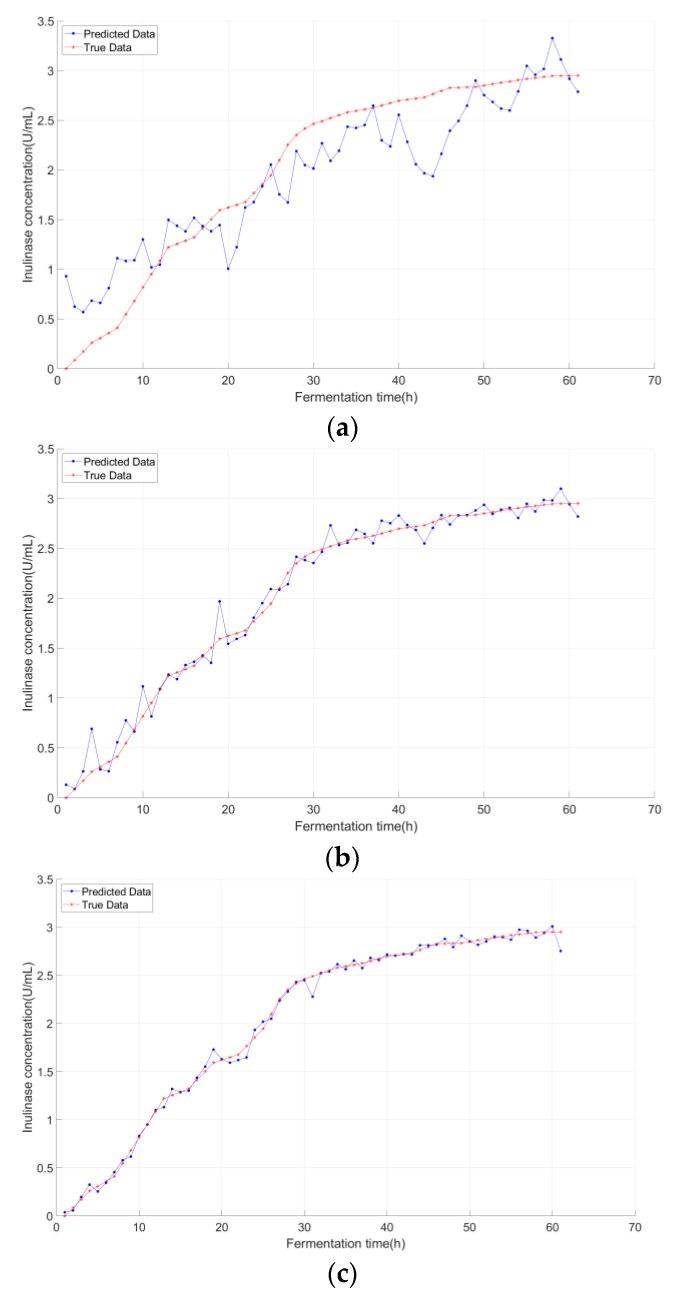
**Inulinase concentration predictions under different modeling approaches:** (**a**) Deep Neural Network (DNN); (**b**) DNN optimized by the conventional Firefly Algorithm (FA-DNN); (**c**) Proposed Model (K-FA-DNN).

**Figure 9:**
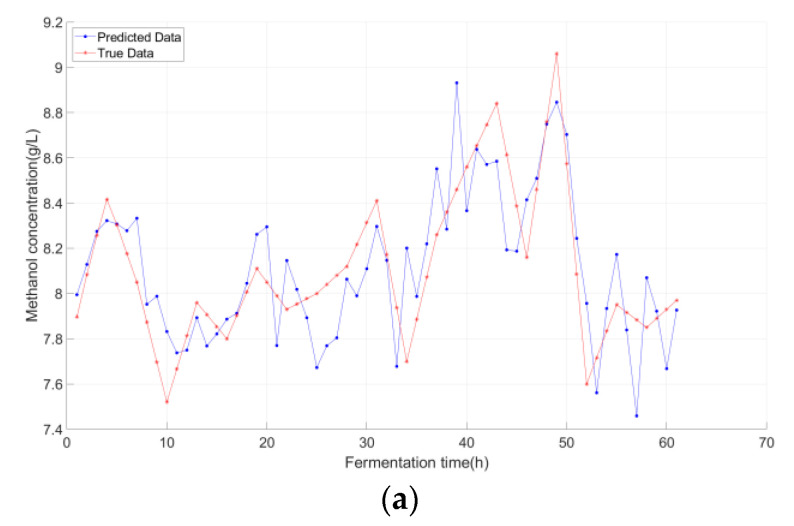

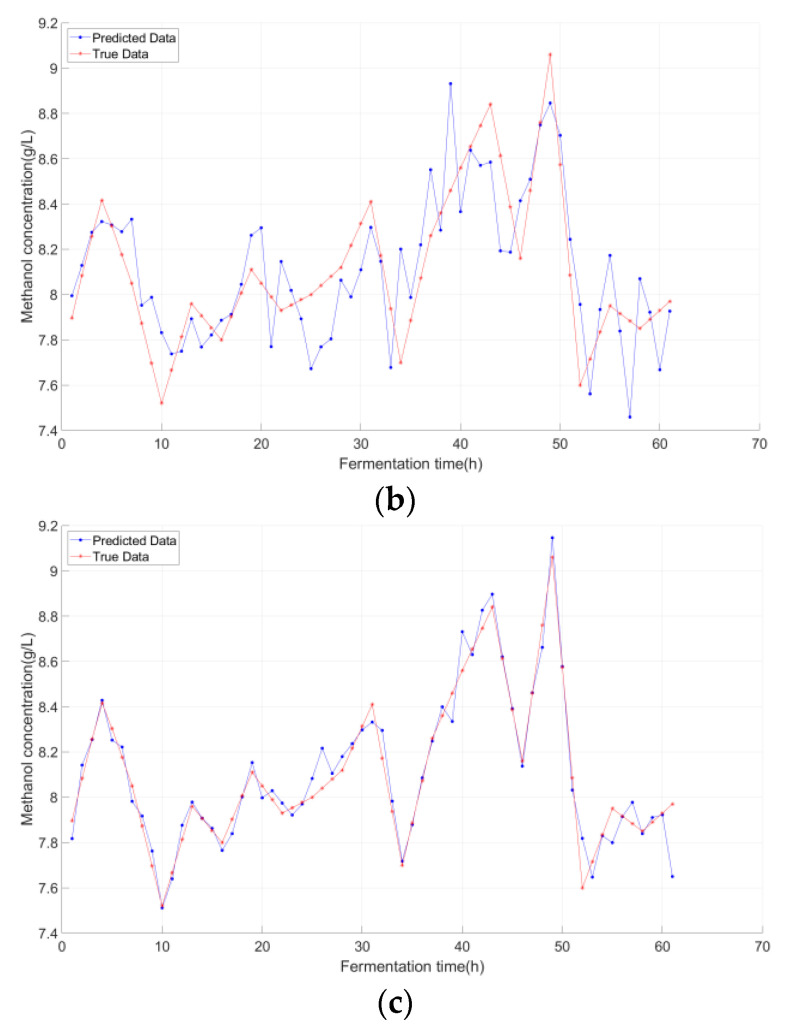
Methanol concentration predictions under different modeling approaches: (**a**) Deep Neural Network (DNN); (**b**) TNN optimized by the conventional Firefly Algorithm (FA-DNN); (**c**) Proposed Model (K-FA-DNN).

Figure 7, Figure 8 and Figure 9 compared the predicted vs. true values of the three key biochemical parameters across the three models, with distinct advantages of K-IFA-DNN model demonstrated:

As evidenced by Figure 7, Figure 8 and Figure 9, the multi-model soft sensor framework—optimized via the enhanced Firefly Algorithm (FA) for deep neural networks (DNNs)—outperformed the single-model traditional DNN in predicting *Pichia pastoris* fermentation variables (biomass, inulinase activity, and methanol concentration). The multi-model architecture significantly improves prediction accuracy by leveraging domain-specific feature partitioning and ensemble learning, demonstrating enhanced robustness and generalization capability.

As clearly demonstrated in Table 1 and Table 2, the prediction errors progressively decreased, and the predictive accuracy improved significantly across models. Notably, the multi-model integration step plays a pivotal role in this enhancement, contributing substantially to the elevated prediction accuracy observed in the final soft sensor framework. These results collectively validate that K-means local partitioning addresses the multi-stage characteristic issue, while the improved firefly algorithm resolved the DNN’s hyperparameter optimization bottleneck—laying a robust foundation for subsequent transfer learning.

### 4.2. Performance Evaluation of Target Domain Model Based on Deep Transfer Fine-Tuning

The framework proposed in this study operates as follows: (1) the Euclidean distance between target samples and each sub-source domain centroid is calculated using Equation (2); (2) the most similar sub-source domain is selected (e.g., the stationary phase sub-domain, with a mean distance $\bar{d}$ = 1.24); (3) the corresponding K-IFA-DNN model is fine-tuned with a learning rate of 1 × 10^−5^ for 50 epochs.

**Figure 10:**
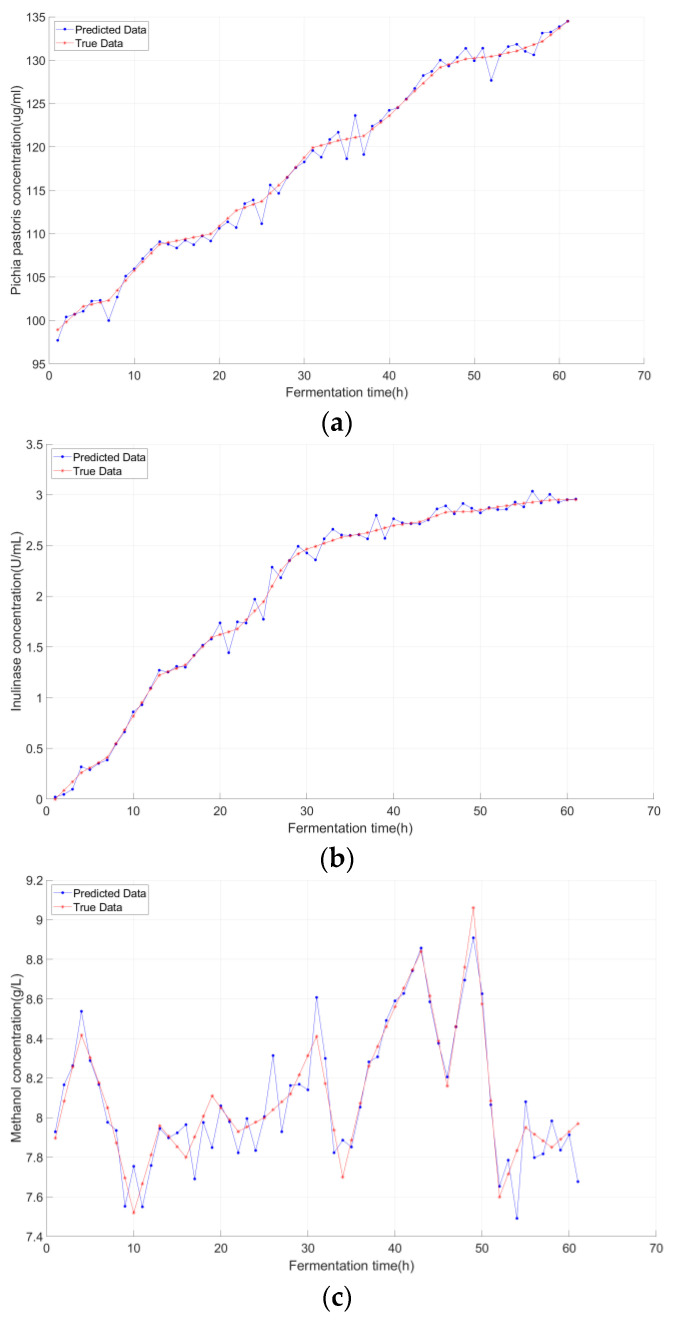
**Structure-Based Transfer Learning Predictions for**: (**a**) *Pichia pastoris* biomass concentration; (**b**) inulinase activity concentration; (**c**) methanol concentration.

The target domain consisted of the third batch of *Pichia pastoris* fermentation data (*n* = 20 samples, no augmentation), which exhibited batch-to-batch heterogeneity (e.g., 10% lower initial biomass, 5% higher methanol feed rate vs. source domain batches). Prediction results for the target domain are visualized in Figure 10.

The proposed Model (Figure 10a) aligns with the true curve (60 h: predicted 134.8 ug/mL vs. true 134.5 ug/mL). By selecting the stationary phase sub-domain (most similar to the target’s growth rhythm), the model only retains relevant features (stationary phase biomass stabilization), and fine-tuning further adapts to the target’s earlier phase transition.

The proposed Model (Figure 10b) accurately captures the elevated peak (60 h: 2.91 U/mL vs. 2.92 U/mL). The stationary phase sub-domain model (pre-trained on source domain product synthesis data) provides a focused feature foundation, and fine-tuning adjusts the output layer weights to match the target’s higher enzyme yield.

Proposed Model (Figure 10c) closely follows the target’s methanol spikes (40 h: 8.59 g/L vs. 8.57 g/L). The selected sub-domain (with similar methanol consumption patterns) enables rapid convergence during fine-tuning, allowing the model to adapt to the higher feed rate.

As clearly demonstrated in Table 3, the proposed model reduced Pichia yeast concentration RMSE to 0.7215, with R^2^ > 0.99, which validated its ability to address batch-to-batch growth rhythm differences. For inulinase concentration (the core target), the proposed model achieved RMSE reduction to 0.0413, with R^2^ approaching 0.997, a level of accuracy that is critical for industrial-scale quality control. Methanol concentration RMSE was reduced to 0.0598, ensuring accurate control of the inducer feed rate.

## 5. Conclusions

This study aimed to address the core challenges of multi-stage characteristics and batch-to-batch data distribution heterogeneity in *Pichia pastoris* fermentation soft sensor modeling—issues that often result in low fitting accuracy and poor generalization of traditional methods. To this end, a novel local transfer modeling framework (K-IFA-DNN-TL) was proposed, integrating K-means-based source domain partitioning, an improved firefly algorithm (IFA)-optimized deep neural network (DNN), and Euclidean distance-guided deep transfer fine-tuning.

The simulation results confirmed the dual advantages of the proposed local transfer framework: ① Local partitioning resolved multi-stage characteristics: By dividing the source domain into stage-specific sub-domains via K-means, the model avoids “average fitting” of global data and accurately captures the nonlinear dynamics of each fermentation stage (e.g., exponential biomass growth, stationary phase inulinase synthesis). ② Improved firefly algorithm optimizes DNN performance: The IFA’s dynamic inertia weight and modified attractiveness function address the conventional FA’s local optima trapping and slow convergence, enhancing the DNN’s ability to fit local stage features. ③ Targeted transfer mitigated batch heterogeneity: Selecting the most similar sub-source domain via Euclidean distance ensures “focused transfer” of relevant features (avoiding negative transfer from irrelevant stages), while deep fine-tuning further adapts the model to batch-specific differences (e.g., earlier phase transitions, higher enzyme yield).

These findings collectively demonstrated that the proposed method outperformed traditional global models and conventional transfer learning approaches, providing a reliable solution for soft sensor modeling in multi-condition *Pichia pastoris* fermentation processes.

Despite its advantages, this study had several limitations that need to be addressed: ① Dependence on manual cluster number selection for K-means: The number of sub-source domains (set to 4 via the elbow method) relies on subjective judgment of the WCSS curve. In cases where fermentation stages overlap (e.g., prolonged transition between exponential and stationary phases), the elbow method may fail to identify the optimal cluster number, leading to suboptimal local modeling. ② Lack of integration with real-time data streams: The current framework used offline preprocessed data for modeling, rather than directly processing real-time sensor data (which may contain noise or missing values). This limited its application in fully automated fermentation control systems.

To address the above limitations and expand the framework’s applicability, future work will focus on the following directions: ① Develop adaptive clustering algorithms: Replace K-means with a fuzzy C-means (FCM) algorithm with adaptive cluster number, which uses fuzzy membership to handle overlapping stages and automatically optimizes the number of sub-domains via the Davies–Bouldin index (DBI). ② Realize online learning for continuous fermentation: Develop a lightweight online learning module that updates the model parameters in real time using streaming data from industrial sensors, enabling adaptive prediction for long-term continuous fermentation processes (e.g., 1000-h fed-batch fermentation).

In summary, the proposed local transfer modeling framework effectively resolved the multi-stage and batch-heterogeneity challenges in *Pichia pastoris* fermentation soft sensor. Its theoretical innovations and practical value provided a new paradigm for soft sensor development in complex biological processes, while the identified limitations and future directions lay the groundwork for further optimization and industrial application.

## Figures and Tables

**Table 1:** RMSE of Prediction Results Under Different Models.

Model	Pichia Yeast Concentration	Inulinase Concentration	Methanol Concentration
DNN	4.8331	0.3721	0.2057
FA-DN	4.4003	0.1191	0.1738
K-IFA-DNN	0.8783	0.0584	0.0785

**Table 2:** *R*^2^ of Prediction Results Under Different Models.

Model	Pichia Yeast Concentration	Inulinase Concentration	Methanol Concentration
DNN	0.7906	0.8388	0.5879
FA-DNN	0.8265	0.9835	0.7060
K-IFA-DNN	0.9931	0.9960	0.9400

**Table 3:** RMSE and *R*^2^ of Target Domain Model.

K-IFA-DNN-TL	Pichia Yeast Concentration	Inulinase Concentration	Methanol Concentration
RMSE	0.7215	0.0413	0.0598
*R* ^2^	0.9914	0.9972	0.9643

## Data Availability

The original contributions presented in this study are included in the article. Further inquiries can be directed to the corresponding author(s).
